# Multiple Mating, Paternity and Complex Fertilisation Patterns in the Chokka Squid *Loligo reynaudii*

**DOI:** 10.1371/journal.pone.0146995

**Published:** 2016-02-12

**Authors:** Marie-Jose Naud, Warwick H. H. Sauer, Niall J. McKeown, Paul W. Shaw

**Affiliations:** 1 School of Biological Sciences, Royal Holloway University of London, Egham, United Kingdom; 2 Department of Ichthyology and Fisheries Science, Rhodes University, Grahamstown, South Africa; 3 Institute of Biological, Environmental & Rural Sciences, Aberystwyth University, Aberystwyth, United Kingdom; University Hospital of Münster, GERMANY

## Abstract

Polyandry is widespread and influences patterns of sexual selection, with implications for sexual conflict over mating. Assessing sperm precedence patterns is a first step towards understanding sperm competition within a female and elucidating the roles of male- and female-controlled factors. In this study behavioural field data and genetic data were combined to investigate polyandry in the chokka squid *Loligo reynaudii*. Microsatellite DNA-based paternity analysis revealed multiple paternity to be the norm, with 79% of broods sired by at least two males. Genetic data also determined that the male who was guarding the female at the moment of sampling was a sire in 81% of the families tested, highlighting mate guarding as a successful male tactic with postcopulatory benefits linked to sperm deposition site giving privileged access to extruded egg strings. As females lay multiple eggs in capsules (egg strings) wherein their position is not altered during maturation it is possible to describe the spatial / temporal sequence of fertilisation / sperm precedence There were four different patterns of fertilisation found among the tested egg strings: 1) unique sire; 2) dominant sire, with one or more rare sires; 3) randomly mixed paternity (two or more sires); and 4) a distinct switch in paternity occurring along the egg string. The latter pattern cannot be explained by a random use of stored sperm, and suggests postcopulatory female sperm choice. Collectively the data indicate multiple levels of male- and female-controlled influences on sperm precedence, and highlights squid as interesting models to study the interplay between sexual and natural selection.

## Introduction

Processes of mate choice, sperm competition and fertilisation dynamics, whether occurring pre- or post-copulation, are important in shaping the evolution of mating systems [1]. Knowledge of observed patterns of mating among individuals is not sufficient to deduce reproductive success, as post-copulatory sperm competition [2] and/or cryptic female choice [3] may affect fertilisation outcomes [4]. Sperm competition has been demonstrated in a variety of organisms including invertebrates [5–7]. Males may employ a number of mechanisms to skew paternity in their favour including removing rival sperm [8–9], blocking the female reproductive tract for subsequent males [10], and chemically reducing female attractiveness to other males [11]. Cryptic female choice mechanisms, so termed because female-controlled influences on paternity are often not externally observable [12], can be passive [13] and/or active [14–16]. Assessing patterns of sperm precedence represents a fundamental first step towards understanding post-copulatory sexual selection and elucidating specific male effects, female effects, or male/female interactions. However, considerable variability in intraspecific precedence patterns may be generated by random sperm mixing [17] and so such stochasticity must be accounted for before patterns can be interpreted in the context of male/female effects. The optimal approach to measure the degree of sperm mixing would be sequential progeny analysis, i.e. by ascribing paternity continuously throughout a fertilisation run [17].

Cephalopods display some of the most complex behavioural adaptations amongst marine invertebrates, particularly in respect of their mating strategies [18]. They are typically highly promiscuous, and females of most species store sperm from multiple males and for long periods of time [19]. Loliginid squid in the genera *Loligo* and *Doryteuthis* (amongst others) engage in complex mating behaviours in mating arenas situated over communal spawning beds on the sea floor [20–24]. Females mate with multiple males over short time periods (minutes to hours) and store spermatozoa in two separate locations in/on their bodies, whilst males transfer discrete packages of sperm (spermatophores) to one or other of these sites according to a mating tactic related to their body size [25,26]. Females pair up and mate in a parallel position with large males (“consorts”), who deposit spermatophores in or around the oviduct opening, and then guard the female until egg laying. Other large males compete for females in agonistic bouts, sometimes displacing the “consort” male, whereas small “sneaker” males attempt extra-pair copulations using quick head-to-head matings when they deposit spermatophores around the seminal receptacle (sperm storage organ) in the buccal area within the arm crown. Fertilisation is external, occurring as eggs are extruded in a long gelatinous string (containing up to several hundred eggs) from the oviduct then drawn forward and held within the female’s arm crown. As the females possess two different sites of sperm storage (on oviduct; on buccal mass plus in sperm receptacle in buccal mass) then sperm competition may occur between males both within and between the different storage sites, each of which is linked to a different mating tactic and sperm/spermatophore morphological differentiation [25]. Pre- and post-copulatory sexual selection are thus likely to be strong in these species [24, 27–29] making them excellent models for studies of sperm precedence

The Chokka squid, *Loligo reynaudii* D’Orbigny, is heavily exploited on its spawning grounds off the coast of South Africa [30]. This species is typical of the loliginid pattern of reproductive and mating strategies described above [21, 22]. Our field observations of *L*. *reynaudii* spawning aggregations indicate that mating and spawning behaviour is very similar to that described quantitatively by Shashar & Hanlon [26] for another day-spawning loliginid squid *Doryteuthis pealei*, and confirm previous observations on *L*.*reynaudii* [21, 22]. Opportunities for females, as well as males, to mate with multiple partners are abundant and appear to be conducted by both sexes. Females have three sources of sperm for fertilisation: in a sperm storage organ within the buccal mass; and in two physically separate sites for sperm deposition which are associated with the two alternative mating tactics employed by large guarding (consort) males (parallel mating, spermatophores deposited on oviduct) and small sneaker males (head-to-head mating, spermatophores deposited around buccal mass). It is known that spermatophore and sperm types present in the two sperm deposition sites in *L*. *reynaudii* match to the pattern reported for *Loligo bleekeri* [25]: “sneaker-type” sperm exclusively in the buccal mass site and “consort-type” sperm exclusively in the oviduct site (Y Iwata, pers.comm.). Eggs are extruded in a string from the oviduct, where they are exposed to sperm from consorts, and then passed through the funnel to be held around the buccal mass within the arm crown, where they are exposed to sperm from sneakers and from the sperm storage organ, before being deposited on the seabed.

An objective of this study was to employ microsatellite-based paternity analysis of wild caught egg strings, incorporating known maternal genotypes, to describe paternity patterns within egg strings. Male mate guarding is suggested to be an adaptive strategy in systems with ‘last male precedence’ [31]. To test this prediction we estimated the proportion of offspring within an egg string attributable to adult males identified as ‘last male consorts’ at the time of sampling. A key feature of this study was that as the females lay multiple eggs in capsules wherein their position is not altered during maturation it was possible to specifically describe the sequence of egg fertilisation/sperm precedence. This permitted us to account for possible random sperm mixing effects and to then describe sperm precedence in the context of sperm competition and/or cryptic female choice.

## Materials and Methods

### Ethics Statement

All sampling was carried out according to local regulations of research ethics and under agreement to WHHS (Rhodes University, South Africa).

### Sample collection and behavioural observations

**Fig 1:**
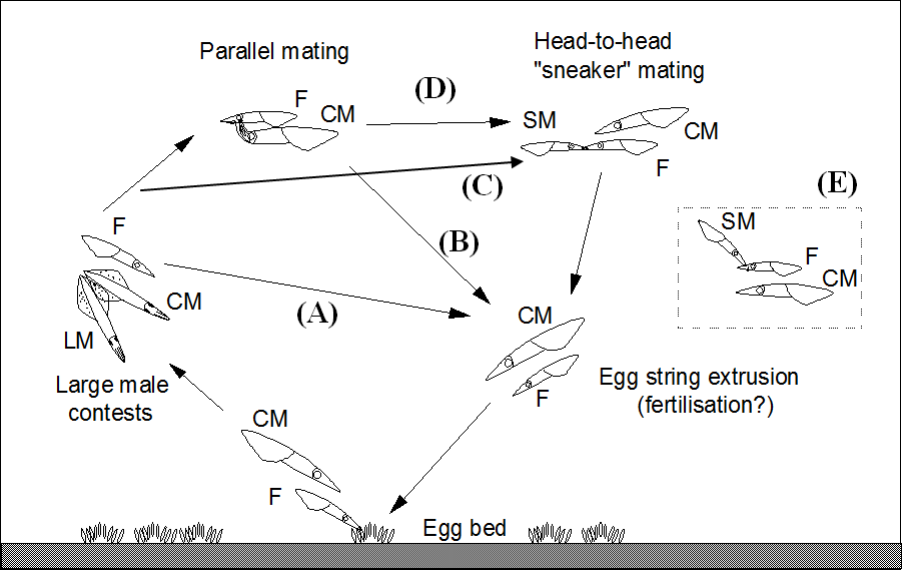
Different behavioural sequences of mating and egg string laying observed for *Loligo reynaudii*: (A) female does not mate with a male; (B) female mates parallel with Consort; (C) female mates head-to-head with “sneaker”; (D) female mates parallel with Consort and head-to-head with “sneaker”; (E) traditional view of sneaker mating, where sneaker rushes in and places spermatophores on female’s arm crown. F = female, CM = Consort (guarding) male, SM = “sneaker” male, LM = large male (agonistic contests between large males for access to the female as her Consort).

Using SCUBA diving mating pairs of *Loligo reynaudii* were observed above the egg bed and then the female (with the fertilised egg string held in her arms) plus guarding (“last male consort”) male captured simultaneously, as described in [29]. In addition some squid mating pairs were followed throughout the mating and egg string laying process (from string deposition to next string deposition) to record behavioural interactions. Due to the extreme difficulty of conducting such a method in the wild (samples collected by SCUBA diving at 25–30m depth, following fast swimming squid, necessity of simultaneous spearing of the correct 2 adults in a mass spawning aggregation plus catching of a floating egg string), it was not possible to reliably combine prolonged behavioural observation of mating with collection of adult/egg samples for targeted females. However, our non-quantitative observations of mating and spawning were able to confirm (after [21, 22, 26]) the range of behaviours exhibited by females that may inform the genotyping results. As depicted in Fig 1 during each cycle of egg string laying one of four sequences may occur before the female extrudes and fertilises an egg string and deposits it on the seabed: A) the female does not mate with any male (although she will have a consort); B) the female mates only in a parallel position with her consort (who places spermatophores on her oviduct); C) the female mates parallel with her consort, then mates head-to-head with a “sneaker” male; D) the female mates head-to-head with a sneaker, but not her consort. The interval between egg string depositions was approximately 3 mins. Consort males are regularly displaced by other large males, who then become the female’s consort.

Samples of female and male tissue were taken and fixed in 95% ethanol. Egg strings were transported to culture facilities, where they were maintained until the embryos had developed to a stage suitable for DNA extraction. All samples were collected from spawning grounds of *Loligo reynaudii* off the Eastern Cape coast of South Africa.

### Microsatellite DNA genotyping

Total DNA was extracted from preserved arm tips of adults or whole embryos using a modified CTAB (2% N-cetyl N,N,N–trimethylammonium bromide) method [32]. Where available at least 28 embryos were tested from each egg string, along with their mating pair adults. To test whether there were any differences in fertilisation success of different males along the length of an egg string, half of the sampled embryos were taken from the proximal (i.e. near the attachment point of the string to the sea bed) and half from the distal ends of the string, with individual positions recorded before removal.

All individuals were genotyped at 3 highly polymorphic species-specific microsatellite loci (Lrey34, Lrey44 and Lrey48; [33]. PCR amplifications were performed in a PTC-200 thermal cycler (MJ Research) and involved an initial denaturation step (94°C for 2 min) followed by 35 cycles of 30s at 92°C, 30s at the locus specific annealing temperature [33] and 30s at 72°C. Reaction mixes contained (total volume of 10 μl): 20 ng of *L*. *reynaudii* DNA, 0.2 μmol of each primer (one primer Cy5-labelled), 0.2 U of *Taq* DNA polymerase (Bioline, UK), 1X the supplied PCR buffer, 1.0 mM (Lrey34) or 1.5 mM (Lrey 44) or 2.5 mM (Lrey48) MgCl_2_, and 0.2 mM dNTPs. PCR products were analysed on 6% denaturing polyacrylamide gels run on an ALFexpressII (Amersham Pharmacia Biotech) automated DNA sequencer with allele sizes inferred against internal standard size markers using FRAGMENT MANAGER version 1.2 (Amersham Pharmacia Biotech).

### Analysis of results

Population allele frequencies at the 3 loci screened were determined from a sample of 67 adults collected from the same site as part of a population survey (see [34]), and from which dataset it was confirmed that none of the loci display linkage disequilibrium or the presence of null alleles. All three microsatellite loci screened were highly polymorphic with 23 (Lvr38), 20 (Lvr44) and 33 (Lvr48) alleles, and expected heterozygosity of 0.93 (Lvr38), 0.93 (Lvr44) and 0.97 (Lvr48).

Using the known maternal genotypes, a multi-locus parsimony approach [35] was used to reconstruct paternal genotypes, and thus assess the number of males contributing fertilisations to each brood. To confirm correct parental assignments for broods, paternal genotypes and offspring assignments were generated using GERUD2.0 [36]. Using the most likely, and conservative, outcome for the paternal genotypes contributing to the offspring array from each egg string, individual offspring were assigned to males. Where more than 1 male contributed to the array, relative fertilisation success was determined and tested for departure from an even ratio (i.e. some males are more successful than others). Using the known position of embryos in the string and their paternal assignment, indications of systematic changes in individual male fertilisation success along the string (i.e. skewed distributions of sired embryos to one end or the other, indicating potential sperm-source switching by the female) were tested against expectations of random distribution. If a significant result was indicated for this last test then all remaining embryos in the string were extracted (after noting their position in proximal, distal or central thirds of the string) and genotyped, then tested again to confirm or reject a skewed distribution.

Tests using the multiple mating simulation programme PrDM [37] indicated that with the loci screened the power to detect multiple paternity within wild broods (one parent known, equal 50:50 or skewed 90:10 paternal contributions) was high (100% and 98% respectively), and similarly the power to provide high parental exclusion probabilities was equally high (with one parent known: Lvr34 = 0.837; Lvr44 = 0.827; Lvr48 = 0.919; combined exclusion probability = 0.998; GERUD2.0). Likewise, simulation studies using GERUDsim 2.0 [36] indicated that with an average sample size of 28 offspring per brood multiple paternity, if present, would be detected in 100% of cases with 50:50, 90:10 or 70:10:10:10 skewed paternal contributions. With very low contributions from some males (84:10:3:3 skew) GERUDsim indicated that the real number of sires would usually be underestimated.

## Results

Of 31 mating pairs sampled 24 were successfully carried through the process, i.e. consisted of genetically matching female and egg string (with consort male), the eggs developed in culture to an embryo stage suitable for DNA extraction, and the embryos and adults were successfully genotyped at the microsatellite loci.

**Fig 2:**
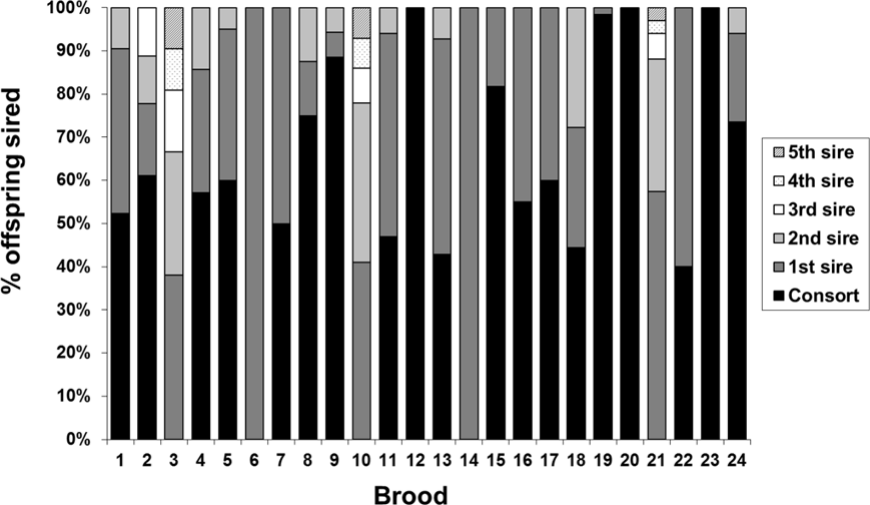
Proportion of eggs sired by each putative father in 24 egg strings of *Loligo reynaudii*.

Reconstruction of paternal male (sire) genotypes indicated multiple paternity within 19 (79.2%) of the 24 egg strings (broods)–see Fig 2. The mean brood size genotyped was 28.4 embryos. The number of inferred sires ranged from 1 to 5 (mean = 2.7, mode = 3) when using both the parsimony approach (number of paternal alleles divided by two) and output from GERUD (Fig 2). Four different patterns of fertilisation along the egg strings were apparent: 1) all embryos fertilised by the same male (“single” sire, frequency = 20.8%); 2) most (> 80%) of the embryos sired by one male and a few sired by one or multiple sires (“dominant” sire, frequency = 12.5%); 3) at least two males sired substantial numbers (>20%) of embryos, and these sires’ embryos were distributed randomly along the egg string (“mixed”, frequency = 45.8%); and 4) at least two males sired substantial numbers (>20%) of embryos, with one sire’s embryos more frequent (than expected at random) at one end of the egg string and the other sire more frequent at the opposite end (“switch”, frequency = 20.8%).

Of the 19 broods displaying multiple paternity there was a significant difference in fertilisation success among the sires represented in 16 (84.2%) cases. The consort male (at the time of sampling) was successful in gaining fertilisations in 19 of 24 (79.2%) broods, and was the most successful sire in 17 (89.5%) of these broods (in 6 cases he is the unique or dominant sire–see Fig 2), with an average fertilisation success of 70.5% (range 43–100%) of the brood. In 5 (20.8%) broods the consort male did not gain any fertilisations: of these 5 broods 2 displayed 100% fertilisation success by an unknown male, and the remaining 3 broods each displayed the maximum number of sires (5) observed in any brood–see Fig 2. When represented (i.e. excluding single-sire broods) the second most successful male achieved an average of 29.0%, the third male 10.9%, the fourth male 7.7% and the fifth male 5.5% of fertilisations. When “non-consort” broods are excluded (i.e. the three broods with 5 sires), then the second most successful male achieved an average of 24.0%, the third male 5.5%, the fourth male 0.6% of fertilisations, and a fifth male was never represented.

Five egg strings (broods 10, 11, 16, 21 and 22 in Fig 2) exhibited a significant skew in the positions, towards the proximal (attachment) or distal ends of the string, of embryos sired by one or both of the most successful males (within that brood). When all embryos within each string were genotyped, four strings (11, 16, 21 and 22, Table 1) still displayed a significantly skewed distribution, although one (brood 22) was of marginal significance (χ^2^, p = 0.04) and was not significant when only the two most successful sires were considered. Of the remaining three significantly skewed broods (11, 16 and 21), all displayed significant departures from expectations across the whole string for all males, and between distal and proximal ends for all sires, the two most successful sires and the most successful sire alone. In all three cases the embryos sired by the most successful male (the consort in 2 of the 3 cases) were skewed towards the distal end of the string (Table 1).

**Table 1:** Distribution of embryos sired by different males in different regions of the egg string, for five *L*. *reynaudii* broods that initial tests indicated to show skewed (non-random) paternity distributions. Regions = Distal, Middle and Proximal thirds of the egg string (All = male % fertilisation success across whole string); n = number of genotyped embryos in string; χ^2^ *P* = significance of departure from uniform distribution.

		% embryos sired by each male				
	Region	C / M1	M2	M3	M4	M5
Brood 10	Distal	60	20	10	5	5
χ^2^ *P* = 0.409	Middle	37	42	0	16	5
	Proximal	25	45	10	5	10
(n = 58)	All	41.4	36.2	6.9	8.6	6.9
Brood 11	Distal	67	33	0		
χ^2^ *P* = 0.001	Middle	55	37	8		
	Proximal	23	69	8		
(n = 116)	All	47.4	47.4	5.2		
Brood 16	Distal	82	18			
χ^2^ *P* < 0.001	Middle	71	29			
	Proximal	7	93			
(n = 43)	All	55.8	44.2			
Brood 21	Distal	72	11	7	5	5
χ^2^ *P* = 0.011	Middle	68	26	2	2	2
	Proximal	30	55	5	5	5
(n = 137)	All	57.7	30.7	4.4	3.6	3.6
Brood 22	Distal	64	36			
χ^2^ *P* = 0.041	Middle	46	54			
	Proximal	93	7			
(n = 41)	All	68.3	31.7			

## Discussion

The evolution of mating systems is a central topic in ecology. Loliginid squid, because of their complex mating behaviours and the possession of alternative male mating tactics and sperm deposition (and so fertilisation) sites [22, 25, 26], offer interesting possibilities to investigate the dynamics of, and evolutionary forces driving, polyandrous mating systems. In a preliminary study of fertilisation patterns in wild *L*. *reynaudii*, Shaw & Sauer [29] found evidence for multiple paternity but also single paternity within broods, skewed fertilisation success among males, and apparently non-random fertilisation patterns within egg strings that could indicate cryptic female choice. The present study represents the largest analysis of squid paternity to date and provides more accurate estimates of parameters vital to understanding of the process of polyandry, specifically the number of males contributing to fertilisation within broods, relationships between male fertilisation success and male mating tactics, and postcopulatory mechanisms that might influence sperm precedence. Such parameters shape the reproductive dynamics of populations and their understanding provides a link between population evolutionary genetics and ecology [38].

The female-guarding behaviour employed by large consort males is a successful sperm competition tactic, as predicted for and observed in other species [39]. Although not as prevalent as reported by Shaw & Sauer [29], and perhaps surprising given the possibilities for multiple paternity in this species, a substantial number (21%) of *L*. *reynaudii* broods show single paternity, and a further 12.5% are sired predominantly by a single male. Of these eight broods, six are sired by last male consorts and the remaining 2 probably also sired by displaced consorts (see below). Despite consorts being successful (most successful male in 89.5% of broods where they achieve fertilisations, 70.5% overall fertilisation success), this tactic is not as successful as observed for other loliginid squid such as *L*. *bleekeri* [24] and *L*. *pealei* [28, 40]. This lower consort mating success in *L*. *reynaudii* most likely results from a reduced ability to defend their access to the female in the very high density (1000s of individuals) mating aggregations, where consorts are displaced more frequently (pers. obs.) than observed for *L*. *pealei* [26].

In 21% of *L*. *reynaudii* broods the sampled consort sired no offspring. Consorts are regularly displaced by other large males close to the egg beds, often when the female is holding an egg string in her arms, just prior to descending to the egg bed, and females do not mate with consorts in every cycle ([22], Naud et al. pers. obs.). We conclude that the absence of last male consort offspring in some broods most likely reflects that these consorts have recently displaced the female’s previous consort and not yet had the opportunity to place their spermatophores in the female’s sperm storage site. It is interesting that among these “non-consort” broods 60% (3 of 5) are the only broods to display offspring from 5 different males and the most even spread of paternities, the remaining two broods being 100% one sire (the “displaced consort”?). An intriguing possibility is that the absence of last male consort paternity within these broods reflects cryptic female choice of sperm. Specifically females could bias paternity by choosing to fertilise eggs soon after mating with a preferred male or before a forced mating with a non-preferred mate (especially in a system with last male sperm precedence and male coercion). Cryptic female choice effected through timing of egg laying has been reported in the soldier fly *Merosargus cingulatus* [41] and is suggested for the Californian market squid *L*. *opalescens* [40]. However, male reproductive success could also be enhanced if males are able to influence the timing of fertilisation such as by manipulative seminal proteins [42].

Aside from the most successful male, usually the consort, there is substantial fertilisation success rate of a second sire represented in most broods displaying multiple paternity. In the present study this male averages 24–29% of fertilisations, compared to 25% in the previous study [29] and lower success rates in other loliginid squid species (23.8% in *L. pealeii–[28];* 23.2 in *L*. *forbesi*–[43]; 5.5% in *L*. *bleekeri*–[24]. Two lines of evidence indicate that this second most successful sire is the sneaker male. First, in laboratory-based squid mating trials sneaker males present in the system achieve these medium-low fertilisation success rates [24, 40]. Second, in the wild squid sneaker males have high mating success rates (e.g. [26]), and in spawning *L*. *reynaudii* females the buccal sperm deposition site always contains large numbers of fresh spermatophores and sperm masses (Naud et al., pers. obs.) that are derived from sneakers only (Y Iwata, pers. comm.). A third, indirect line of argument for the second most successful sire being a sneaker is: how could such a distinct mating strategy persist without substantial reproductive success? The sneaker tactic is a highly evolved alternative mating strategy involving distinct morphological and behavioural characteristics representing individual specialism [25]. To further explore the co-evolution of consort and sneaker strategies, the identity of the “second male” requires direct testing by matching wild-caught sneaker genotypes to buccal mass sperm and offspring in egg strings.

As predicted from observed behaviour, and in line with other cephalopod species (e.g., [28, 44, 45]), 79% of female *L*. *reynaudii* tested displayed multiple paternity within their broods, with sperm from up to five different males used to fertilise eggs within a single brood, indicating high rates of multiple mating by females and use of stored sperm. Four apparent patterns of proportional skew in paternity within egg strings are reported here (single sire; dominant sire with low frequencies of 1–3 further sires; mixed paternity with 2 high frequency sires and 1–3 low frequency sires, and including the special case of a distinct switch in siring along the string). Using simulations, Harvey & Parker [17] demonstrated that a range of paternity skews can be derived from random patterns of sperm mixing that are independent of any selective drivers, which suggests that the patterns observed in *L*. *reynaudii* could result from random biomechanical mixing of sperm. However, as described above, in *Loligo* sperm are released onto the eggs from at least two physically separate sources on the female, and not into a single fertilisation site as present in most polyandrous species and as modelled by [17]. The process of extrusion of squid eggs, and their coating in jelly and wrapping into egg strings, is a complex one [46], but which occurs before the eggs leave the oviduct. After extrusion the egg string is exposed to clouds of free-swimming sperm, first those released from consort spermatophores around the oviduct and second those released from sneaker spermatophores around, and from the sperm storage organ within, the buccal mass. Therefore, while there is likely to be mixing of sperm within the sperm storage organ and it cannot be ruled out that there may be some mixing of ‘sperm clouds’ derived from the three sperm sites, the sequential pattern of egg string movement is expected to impart a deterministic influence on fertilisation, with fertilisation access granted to consort sperm first. This is supported by the realised paternity results reported here and by [29] demonstrating general precedence of consort sperm, and points to sperm placement by males (consort and sneakers exhibiting characteristic sperm placement patterns) as a sperm competition tactic.

After copulation, females could exercise postcopulatory choice of stored sperm [3], though this has not yet been tested in squid and is difficult to demonstrate. For example, it has been speculated that females may influence consort sperm residency through mantle flushing, or perhaps removing spermatophores using their dextrous arms. It has also been suggested that paternity may be influenced by the way females hold their egg strings [26,47]. In the context of female postcopulatory sperm choice the significant spatial patterning of paternity within egg strings is intriguing. In this study 21% of multiply sired broods displayed a significant spatial patterning of sires’ offspring along the egg string with the overall proportion increasing to 29% of multiply sired broods if the preliminary results of [29] are included. As far as we are aware this is the first report of spatial patterning of offspring by sire within a single egg case, whereas other studies (where spatial arrangements have been investigated) have shown random offspring distributions (e.g. [48]). While the proportional skews reported may be shaped by deterministic and/or stochastic factors associated with the sequential encounter of the egg string with sperm from different sources no spatial patterning is expected as the entire egg string is exposed to sperm. The spatially skewed offspring distribution could result from egg strings that are only partially extruded from the oviduct so that the exposed section is accessible to (consort) sperm only. When fully extruded and transferred to the buccal area, exposure to sneaker/receptacle sperm could generate skews of consort offspring at one end and sneaker offspring at the other: the consistent skewing of consort offspring to the distal end of the egg string supports this hypothesis. Why this skewing occurs in some egg strings and not others may be a random physical process or may represent active female control of this process, i.e. cryptic female sperm choice [3]. Together with the correlation of use of receptacle stored sperm with “non-consort” broods, the systematic skewing of individual siring along egg strings suggests the possibility of cryptic female sperm choice in wild *L*. *reynaudii*.

While caution must always be exercised in making strong claims about patterns and mechanisms of sperm precedence [49], this study indicates multiple levels of sperm competition and female choice and has generated a number of specific hypotheses that could be tested by future field and laboratory based research programs. The proportion of multiple mating females and detected incidents of multiple paternity reported here must also be considered minimum estimates (e.g. sampling more eggs could reveal more fathers). As these squid display little pre-mating male-female association and no post-spawning parental care, direct benefits of mate choice (other than sperm replenishment) are unlikely to be important and the evolutionary basis for such extreme polyandry is likely to be linked to indirect benefits such as genetic diversity among offspring or the “sexy sons” hypothesis of sexual selection [50]. Loliginid squid have approximately annual semelparous life cycles with post-spawning mortality, and display large and unpredictable fluctuations in population size [51], where individual-level and population-level flexibility to respond to environmental variation would be advantageous. Maximising genetic diversity amongst offspring would appear to be an obvious driver of polygamy in these species. *L*. *reynaudii* thus represents an interesting model for future studies of the interplay between natural and sexual selection.
